# Effects of Xylo-Oligosaccharides on Growth and Gut Microbiota as Potential Replacements for Antibiotic in Weaning Piglets

**DOI:** 10.3389/fmicb.2021.641172

**Published:** 2021-02-25

**Authors:** Yuxia Chen, Yining Xie, Ruqing Zhong, Lei Liu, Changguang Lin, Lin Xiao, Liang Chen, Hongfu Zhang, Yves Beckers, Nadia Everaert

**Affiliations:** ^1^State Key Laboratory of Animal Nutrition, Institute of Animal Science, Chinese Academy of Agricultural Sciences, Beijing, China; ^2^Precision Livestock and Nutrition Unit, Gembloux Agro-Bio Tech, TERRA Teaching and Research Centre, Liège University, Gembloux, Belgium; ^3^School of Life Sciences and Engineering, Southwest University of Science and Technology, Mianyang, China; ^4^Institute of Animal Husbandry and Veterinary Medicine, Fujian Academy of Agriculture Sciences, Fuzhou, China; ^5^Shandong Longlive Bio-Technology Co., Ltd., Yucheng, China

**Keywords:** xylo-oligosaccharides, growth performance, gut microbiota, antibiotics, weaned piglets

## Abstract

Xylo-oligosaccharides (XOS) is a well-known kind of oligosaccharide and extensively applied as a prebiotic. The objective of this study was to investigate the effect of XOS supplementation substituting chlortetracycline (CTC) on growth, gut morphology, gut microbiota, and hindgut short chain fatty acid (SCFA) contents of weaning piglets. A total of 180 weaned piglets were randomly allocated to three treatments for 28 days, as follows: control group (basal diet, CON), basal diet with 500 mg/kg (XOS500) XOS, and positive control (basal diet with 100 mg/kg CTC). Compared with the CON group, the piglets in the XOS500 group improved body weight (BW) on days 28, average daily gain (ADG) and reduced feed: gain ratio during days 1–28 (*P* < 0.05). The XOS500 supplementation increased Villus height and Villus height: Crypt depth ratio in the ileum (*P* < 0.05). Villus Height: Crypt Depth of the ileum was also increased in the CTC treatment group (*P* < 0.05). Meanwhile, the XOS500 supplementation increased significantly the numbers of goblet cells in the crypt of the cecum. High-throughput 16S rRNA gene sequencing revealed distinct differences in microbial compositions between the ileum and cecum. XOS500 supplementation significantly increased the bacterial diversity. However, CTC treatment markedly reduced the microbial diversity (*P* < 0.05). Meanwhile, XOS500 supplementation in the diet significantly increased the abundance of *Lactobacillus* genus compared to the CON and CTC group in the ileum and cecum (*P* < 0.01), whereas the level of *Clostridium_sensu_stricto_1*, *Escherichia-Shigella*, and *Terrisporobacter* genus in the XOS500 group were markedly lower than the CON and CTC group (*P* < 0.05). In addition, dietary supplementation with XOS500 significantly increased the total short-chain fatty acids, propionate and butyrate concentrations and decreased the acetate concentration compared to the CON group in the cecum (*P* < 0.05). In summary, dietary supplemented with XOS500 could enhance specific beneficial microbiota abundance and decrease harmful microbiota abundance to maintain the structure of the intestinal morphology and improve growth performance of weaned piglets. Thus, XOS may potentially function as an alternative to in-feed antibiotics in weaned piglets in modern husbandry.

## Introduction

Weaning piglets in modern swine industry are often challenged by post-weaning stresses including dietary, social, and environmental changes. These stresses result in increasing disease and mortality risks, reducing growth rates and rising impairment of the intestinal microbiota. Every year, 17% of piglets born in Europe died during weaning due to opportunistic pathogen infection (Gresse et al., 2017). China is the biggest pork producer and consumer in the world but about 24 million weaning piglets every year die from diarrhea due to inappropriate treatment during weaning (Wang T. et al., 2019).

Antibiotics were widely used in weanling piglets to promote animal growth and prevent infections (Cromwell, 2002; Wijtten et al., 2011; Hu et al., 2020). However, the addition of antibiotics in feed can result in changes in the intestinal microbiota due to its broad-spectrum antibacterial activity (Neuman et al., 2018). In addition, antibiotics resistance and antimicrobial residues has become a major threat in treating pathogenic bacterial infections (Toutain et al., 2016). For example, apramycin sulfate was widely used in China to prevent piglet diarrhea, however, taking apramycin might cause cross-resistance of apramycin/gentamicin in *Escherichia coli* and *S. enteritidis* (Herrero-Fresno et al., 2016). For these reasons, the European Commission decided to ban the use of antibiotics as feed additives since January 2006 due to the risk of spreading antibiotic resistance (Smith et al., 2010). Other countries are also trying to gradually reduce or forbid use of feed antibiotics. For instance, the use of colistin sulfate as feed additives in animal diets has been banned in China since April, 2017 and India since July, 2019 (Wang Y. et al., 2020). Therefore, it is urgent to develop novel alternatives to antibiotic feed additives.

Recently, several alternatives to antibiotics were reported to maintain swine health and improve growth performance, including probiotics, prebiotics, acidifiers, and essential oils (Gresse et al., 2017; Wang W. et al., 2019). Prebiotics are defined as a substrate that is selectively utilized by host microorganisms conferring benefits upon host health (Gibson et al., 2017). Callaway et al. (2008) showed that prebiotics are a preferable alternative to antibiotics. Commercially available prebiotics mainly include xylo-oligosaccharides (XOS), fructo-oligosaccharides (Mikkelsen et al., 2003), inulin (Mair et al., 2010), mannan-oligosaccharides (Zhao et al., 2012), galacto-oligosaccharides (Alizadeh et al., 2015), and transgalacto-oligosaccharides (Mikkelsen and Jensen, 2004). XOS are sugar oligomers made up of 2–6 xylose units linked through β-(1→4)-linkages (Samanta et al., 2015). XOS has been demonstrated to improve animal health, growth performance, and enhance the role of endogenous beneficial microbiota, such as *bifidobacterium* and lactic acid bacteria in the gut (Gobinath et al., 2010; De Maesschalck et al., 2015; Yang et al., 2015). However, the effect of XOS as antibiotic substitution in weaned piglets has not been reported until now. Therefore, this study aimed to investigate the effects of dietary supplementation with XOS as potential replacements for antibiotic on the growth, gut morphology, gut microbiota, and hindgut SCFA contents of weaned piglets.

## Materials and Methods

### Animals and Experimental Design

This study was approved by the Animal Welfare Committee of Institute of Animal Sciences, Chinese Academy of Agriculture Sciences (IASCAAS). All animal treatments in this study were performed according to the guidelines of the Animal Care and Use Committee of the Chinese Academy of Agriculture Sciences (CAAS). Humane animal care was practiced throughout the experiments and every effort was made to minimize suffering for piglets (ethics approval code: IAS2019-34).

A total of 180 healthy weaned piglets (Duroc × Landrace × Large White, weaned at 28 d of age) with an average initial body weight (BW) of 8.84 ± 0.25 kg, were randomly assigned to three treatments based on the BW and sex. The control group with basal diet without any antibiotics or prebiotics (CON), and the antibiotic group with basal diet supplemented with 100 mg/kg pure chlortetracycline (CTC) were attributed as the positive control group. The XOS treated group piglets were fed 500 (XOS500) mg/kg corncob-derived XOS (Longlive Biotechnology Co., Ltd., Shandong, China). This XOS has a purity of 95% a degree of polymerization (DP) 2–7 and is formed by xylose residues linked through β-(1,4)-linkages monomeric units. Prior to the trial, no clinical signs of diarrhea or other diseases were observed in the piglets. All pigs here had similar husbandry practices. Each treatment had four replicated pens with 15 pigs per pen. All diets were formulated to provide all of the nutrients to meet NRC requirements in 2012 (Table 1). The relative humidity and temperature of the piglet house were monitored at 60–65% and 25–28°C, respectively. Piglets were allowed *ad libitum* access to feed and water throughout the experiment for 28 days.

**TABLE 1:** Composition and nutrient levels of basal diets (basis).

**Item**	**CON**
**Ingredients (%)**	
Corn	59.00
Soybean meal	18.40
Fermented soybean meal	5.00
Fish meal	3.00
Soybean oil	2.50
Dried whey	5.00
Sugar	2.00
Glucose	2.00
CaHPO_4_	0.50
Limestone	0.50
Salt	0.30
Lysine HCl	0.40
Met	0.10
Thr	0.10
Choline chloride	0.10
Anti-mildew agent	0.10
Premix	1.00
**Nutrient level**	
Dry matter (%)	87.80
Crude protein (%)	20.00
Crude fiber (%)	1.60
Neutral detergent fiber (%)	22.90
Acid detergent fiber (%)	3.70
Gross energy (cal/g)	4,563.00

*The premix provided the following per kg of diet: vitamin A, 13500 IU; vitamin D3, 2925 IU; vitamin E, 45 mg; vitamin K3, 36.75 mg; vitamin B1, 6.75 mg; vitamin B2, 11.25 mg; vitamin B6, 7.2 mg; vitamin B12,0.054 mg; nicotinamide, 54 mg; calcium pantothenate, 15.75 mg; folic acid, 1.8 mg; biotin, 0.342 mg; Fe, 140 mg; Cu, 20 mg; Zn, 100 mg; Mn, 30 mg; I, 0.4 mg; Se, 0.4 mg.*

### Sample Collection

At 28 days, six piglets from each group were chosen randomly and euthanized aseptically. Afterward, the entire intestine was removed from each pig. Segments of the ileum and cecum flushed with saline were collected. These intestinal segments were immediately fixed in 4% paraformaldehyde solution and then embedded in paraffin for morphological examination. The luminal digesta of the ileum and cecum was collected aseptically into sterile plastic containers and stored at −80°C until processing.

### Morphological Examination

PAS staining was performed according to standard protocols (Shatos et al., 2003). Paraformaldehyde-fixed ileum and cecum segments taken were then dehydrated with ethanol, embedded in paraffin, and sectioned (5 μm). After dewaxing and immediately washing with distilled water for 1 min, the specimens were immersed in 0.5% periodate solution (Sigma Co.) for 5 min at room temperature in the dark. Afterward, sections were immediately washed (30 s × 2) and soaked in Schiff’s solution at 37°C. After 60 min, sections were washed twice with a sulfuric acid solution then quickly rinsed with distilled water. The subsequent steps followed the routine protocols of the laboratory. The sections were examined using light microscopy. The villus length, crypt depth and the numbers of goblet cells were measured by random measurement of 10 villi and 10 measurements of the crypt per section using DS-U3 (Nikon, Japan).

### Genomic DNA Extraction

For 16S rDNA sequencing, six individuals from eight slaughtered piglets in every group were selected randomly (*n* = 6). Microbial DNA of digesta samples of the cecum was extracted using the E.Z.N.A.^®^ soil DNA Kit (Omega Bio-tek, Norcross, GA, United States) according to the manufacturer’s protocols. The final DNA concentration and purification were determined by NanoDrop 2000 UV-vis spectrophotometer (Thermo Scientific, Wilmington, NC, United States), and DNA quality was evaluated on 1% agarose gels.

### Illumina Mi-seq Sequencing

To analyze the taxonomic composition of the bacterial community, the V3–V4 hypervariable regions of the bacterial 16S rRNA gene were amplified with primers 338F (5′-ACTCCTACGGGAGGCAGCAG-3′) and 806R (5′-GGACTACHVGGGTWTCTAAT-3′) by thermocycler PCR system (GeneAmp 9700, ABI, United States). The PCR reactions were performed with the following program: an initial denaturation at 95°C for 3 min, 27 cycles of 30 s at 95°C, annealing 55°C for 30 s, and 45 s for elongation at 72°C, and a final extension at 72°C for 10 min and held at 4°C. PCR reactions were performed in triplicate with a final volume of 20 μL mixture containing 4 μL of 5 × FastPfu Buffer, 2 μL of 2.5 mM dNTPs, 0.8 μL of each primer (5 μM), 0.4 μL of FastPfu Polymerase and 10 ng of template DNA. The PCR products were extracted using electrophoresis on 2% agarose gels and further purified with an AxyPrep DNA Gel Extraction Kit (Axygen Biosciences, Union City, CA, United States) and quantified using QuantiFluor^TM^-ST (Promega, United States). Purified amplicons were pooled in equimolar and paired-end sequenced (2 × 250 bp) on an Illumina MiSeq platform (Illumina, San Diego, CA, United States) according to the standard protocols. Majorbio Bio-Pharm Technology Co., Ltd. (Shanghai, China) carried out the sequencing.

### Bioinformatics Analysis

Raw read quality was quality-filtered using the QIIME (version 1.9.0^1^) software package according to the following criteria: (i) the reads were clipped with an average quality score <20 over a 50-bp sliding window. (ii) sequences whose overlap being longer than 10 bp were merged according to their overlap with mismatch no more than 2 bp. (iii) reads with more than two nucleotide mismatches in the primer, any mismatch in barcode, or ambiguous nucleotides were removed. The clean reads were compared with the reference database using the UCHIME algorithm to detect chimera sequences (Edgar et al., 2011). Operational taxonomic units (OTUs) were clustered with 97% similarity level using UPARSE (version 7.1^2^) with a novel “greedy” algorithm that performs chimera filtering and OTU clustering simultaneously. The taxonomy of each 16S rRNA gene sequence was analyzed by RDP Classifier algorithm^3^ against the Silva (SSU123) 16S rRNA database using a confidence threshold of 70%. α-diversity indices including Chao1 value and Shannon index were determined by the Mothur software package (Schloss et al., 2009). β-diversity was investigated with QIIME using principal coordinate analysis (PCoA) based on the Bray Curtis distance matrix.

### Absolute Quantification of Cecal Specific Bacteria by qPCR

The designs of primers used for absolute quantification of cecal specific bacteria *Lactobacillus*, *Clostridium_sensu_stricto_1*, and *Terrisporobacter via* qPCR are shown in Supplementary Table 1. Tenfold serial dilutions of the genomic DNA of cecal samples from 10^–1^ to 10^–6^ were subjected to qPCR to generate a standard curve. The qPCR assay of standards, samples, and no-template control was performed in triplicate on an Applied Biosystems 7500 Real-Time PCR System (Thermo Fisher Scientific, United States) with a 96-well format.

The 20 μL reaction mixture contained 10 μL of SYBR Premix Ex Taq (Tli RNase H Plus; 2 × concentration) from TaKaRa (Shiga, Japan); 0.5 μL of each 10 μmol/L primer; 7 μL of sterile DNase-free water; and 2 μL of DNA. The PCR was performed under the following conditions: 95°C for 30 s, followed by 40 cycles of 5 s at 95°C, 40 s at 60°C, and 20 s at 72°C. The fluorescence signal was acquired following the 72°C extension phase of each cycle. Melting curve analysis was performed to check the specificity of the products followed by a cooling step performed at 95°C for 10 s, 60°C for 60 s, and 95°C for 15 s (ramp rate of 0.05°C/s). PCR products that had been resolved on a 3% agarose gel were checked after ethidium bromide staining to confirm the specificity of the amplification. Data were analyzed with ABI 7500 Real-Time PCR software version 2.0.5 using the second derivate maximum method, which calculates PCR efficiency in accordance with Pfaffl (2001).

### Composition of Short Chain Fatty Acid in the Cecum

The concentration of short chain fatty acids (SCFAs) in the cecum was measured using the method described by Erwin et al. (1961) with modifications. In brief, about one gram of cecal contents were thoroughly mixed with 10 ml distilled water, incubated at 4°C for 24 h and centrifuged at 10,000 *g* for 10 min at 4°C. 0.9 ml of supernatant was mixed with 0.1 ml 25% (v/v) metaphosphoric acid and kept in the ice bath for at least 30 min. Then, the sample was centrifuged at 10,000 *g* for 10 min at 4°C and 800 microliters of the sample were injected for analysis on an Agilent 6890N GC (Palo Alto, CA, United States).

### Statistical Analysis

To compare differences among different treatments, all data were subjected to ANOVA using the MIXED procedure of SAS (SAS9.4, Cary, NC, United States). Treatment means were calculated using the LSMEANS statement and means were separated using the PDIFF option. Least square means were compared using the Tukey–Kramer adjustment. The differences were considered to be statistically significant if *P* ≤ 0.05 or 0.001 < *P* ≤ 0.01 and were considered extremely significant if the *P* < 0.001. While 0.05 < *P* ≤ 0.1 was considered as having a trend of difference.

## Results

### Growth Performance

**FIGURE 1:**
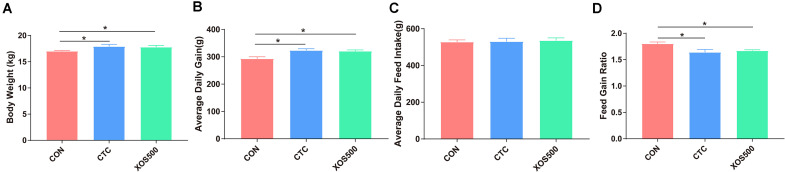
Effect of dietary treatments on growth performance in weaned piglets. **(A)** Body weight, **(B)** average daily gain, **(C)** average daily feed intake, and **(D)** feed gain ratio. CON, control; CTC, chlortetracycline; XOS500, 500 mg/kg XOS. *P ≤ 0.05.

To evaluate the effect of XOS on growth performance of weaned piglets, the BW and average daily feed intake (ADFI) were measured, and average daily gain (ADG) and feed: gain ratio (F:G) were calculated (Figure 1). Piglets in the CTC group and XOS500 group had higher BW and higher ADG during days 1–28 than those in the CON group (*P* < 0.05). Compared with the CON group, the CTC and XOS500 groups had also significantly better F:G during days 1–28 (*P* < 0.05). However, there was no significant difference between the CTC and XOS500 group for growth performance indices of weaned piglets. No difference in ADFI was observed during different dietary groups.

### Effects of Dietary Treatments on Ileal and Cecal Morphology

**FIGURE 2:**
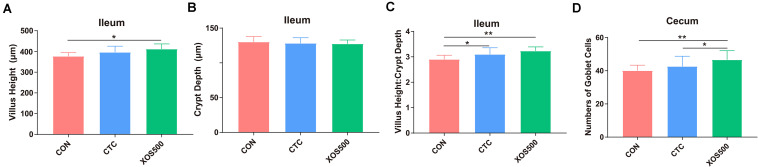
Effect of dietary treatments on histological morphology in the ileum and cecum of weaned piglets. **(A)** Villus height (ileum), **(B)** crypt depth (ileum), **(C)** villus height: crypt depth (ileum), and **(D)** numbers of goblet cells in the crypt of cecum. CON, control; CTC, chlortetracycline; XOS500, 500 mg/kg XOS. *P ≤ 0.05; **0.001 < *P* ≤ 0.01.

The effects of dietary treatments on intestinal characteristics are shown in Figure 2. The XOS500 supplementation increased Villus Height and Villus Height: Crypt Depth Ratio in the ileum (*P* < 0.05). Villus Height: Crypt Depth Ratio was also increased in the ileum as the CTC treatment (*P* < 0.05). Meanwhile, the XOS500 supplementation increased significantly the numbers of goblet cells in the crypt of the cecum (*P* < 0.05) compared with the CTC and CON group.

### DNA Sequence Analysis and Quality Filtering

A total of 1,857,463 valid sequences from 36 ileal and cecal samples remained after chimeras were filtered out and low-quality sequences were removed. Among the high-quality sequences, about 99.98% were longer than 400 bp, with an average of 433 bp. Results showed that the all Good’s coverage was >0.99, implying that most of the microbial diversity within the samples had been sufficiently captured.

### Comparison Between the Ileal and the Cecal Microbiota

**FIGURE 3:**
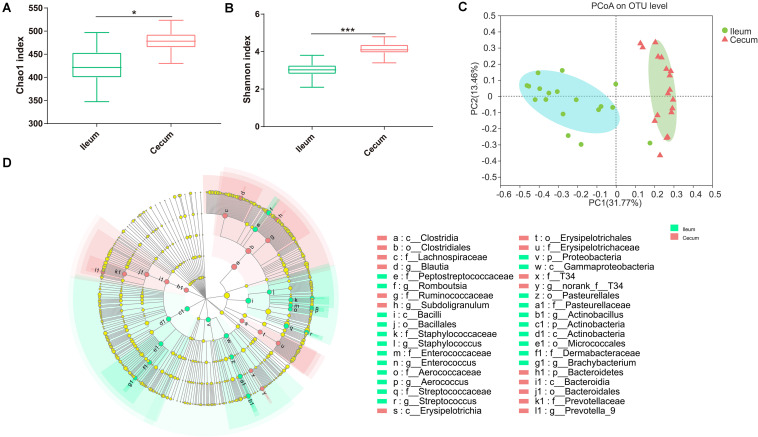
Differences between ileal and cecal microbiota of weaned piglets with supplementation XOS. Alpha diversity for ileal and cecal bacteria, for the observed **(A)** Chao1 index and **(B)** Shannon index. **(C)** PCoA analysis of OTUs indicates that the bacterial profile differed strongly according to sampling site (*R* = 0.71, *P* = 0.001). **(D)** Taxa significantly associated with ileal communities (red) vs. cecal communities (blue), shown in circular cladogram based on the LDA. The diameter of each circle is proportional to the abundance of the taxon. Biomarker taxa are heighted by colored circles and shaded areas. The yellow nodes indicate taxa that were not significantly differentially represented. Only differentially abundant taxa at the genus or higher taxonomic ranks were indicated. *P ≤ 0.05; ***P ≤ 0.001.

According to the Chao1 index and Shannon index (*P* < 0.01), there were significant differences in microbiota richness and diversity in the cecal samples compared with those in corresponding ileal samples (Figures 3A,B), indicating that the cecal microbiota was more diversified than the ileal microbiota. Furthermore, PCoA plots using the Bray–Curtis matrix distances, where bacterial communities clustered by the intestine, clearly showed the distinct bacterial community structure between the ileum and cecum (*R* = 0.70, *P* = 0.001, Figure 3C). All differential bacteria were shown in the cladogram of LEfSe in the ileum and the cecum. The circles from inner to outer represent distinct bacteria from phylum to genus levels, respectively. The yellow dots inserted in the circle suggest no significant difference in bacteria among different dietary treatments. LEfSe results showed that 38 bacterial clades at all taxonomic levels were differentially abundant (LDA > 4.0) between the ileal and cecal microbiota (Figure 3D).

### Effects of Dietary Treatments on the Ileal Microbiota

**FIGURE 4:**
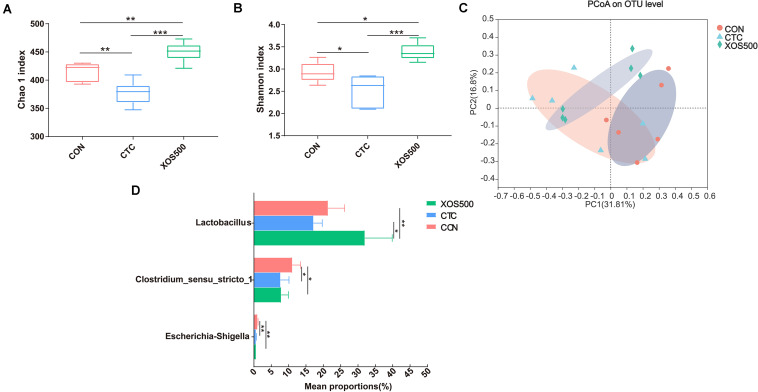
Effect of dietary treatments on ileal microbiota diversity and composition of weaned piglets. **(A)** Chao1 index, **(B)** Shannon index, and **(C)** the beta-diversity of ileal microbiota of piglets. Principal coordinates analysis (PCoA) was used to compare the composition of ileal microbiota in different diet treatments group using Bray–Curtis. **(D)** Kruskal–Wallis *H* test bar plot showed the major different ileal bacterial genus during the different treatment groups. CON, control; CTC, chlortetracycline; XOS500, 500 mg/kg XOS. *P ≤ 0.05; **0.001 < *P* ≤ 0.01; ***P ≤ 0.001.

Alpha diversity was evaluated in this study by analyzing the Chao1 index and Shannon index. Both the Chao 1 and Shannon index in the CTC group was significantly lower than the CON group. However, XOS500 supplementation significantly increased the ileal bacterial index of observed-species, the Chao1 and Shannon index (Figures 4A,B). Beta diversity was assessed by using the Bray–Curtis distance matrices and principal component analysis (PCoA). It was clear that the microbiota in the XOS500 group could separate from the CON group. The CTC group microbiota did not separate from the CON group community (Figure 4C). At the phylum level, the dominant phylum was *Firmicutes* in the ileum in each group (Supplementary Figure 1). At the genus level, the predominant genus was *Lactobacillus*. The relative abundance of *Lactobacillus* in the XOS500 group was significantly higher than in the CON and CTC group (*P* < 0.01), while the relative abundance of *Clostridium_sensu_stricto_1* and *Escherichia-Shigella* were remarkably reduced in the CTC and XOS500 group (Figure 4D).

### Effects of Dietary Treatments on the Cecal Microbiota

**FIGURE 5:**
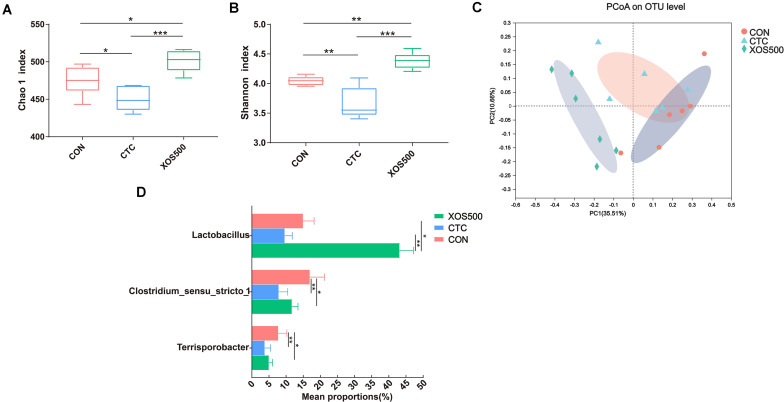
Effect of dietary treatments on cecal microbiota diversity and composition of weaned piglets. **(A)** Chao1 value, **(B)** Shannon index, and **(C)** the beta-diversity of cecal microbiota of piglets. Principal coordinates analysis (PCoA) was used to compare the composition of cecal microbiota in different diet treatments group using Bray–Curtis. **(D)** Kruskal–Wallis *H* test bar plot showed the major different cecal bacterial genus during the different treatment groups. CON, control; CTC, chlortetracycline; XOS500, 500 mg/kg XOS. *P ≤ 0.05; **0.001 < *P* ≤ 0.01; ***P ≤ 0.001.

The Chao1 index and Shannon index were calculated using the OTU counts for each group and then compared among three dietary treatments (Figures 5A,B). The results showed that the Chao1 index and Shannon index in the XOS500 group were significantly higher than the CON and CTC group in this study. To determine similarities between microbial communities, we compared the Bray–Curtis distance of the cecum content samples from the three dietary treatments (Figure 5C). It was clear that the microbiota in the XOS500 group was separated from the CTC and CON group. The first axis of the PCoA explained 35.51% of the variation in bacterial diversity while the second axis explained 10.66%. No significant differences were observed with respect to the relative abundances of bacterial phyla in the cecum during these groups (Supplementary Figure 2). At the genus level, the relative abundances of *Lactobacillus* in the XOS500 group were significantly higher than the CON and CTC groups. Meanwhile, the CTC supplementation could remarkably reduce the relative abundances of *Lactobacillus* compared to the CON group. However, the piglets in the XOS500 and CTC group showed a lower relative abundance of *Clostridium_sensu_stricto_1* and *Terrisporobacter* in comparison to the CON group (Figure 5D). The butyrate-producing genus *Blautia* and *Faecalibacterium* in the XOS500 group displayed an increasing trend compared to the CTC and CON group (0.05 < *P* < 0.1), while no significant differences were observed among the three groups (Supplementary Figure 3).

### Absolute Quantification of Cecal Specific Microbiota by qPCR Assays

**FIGURE 6:**
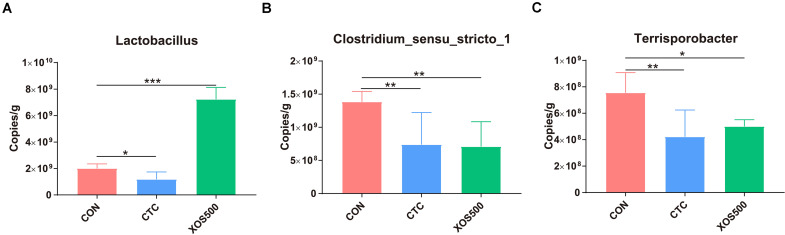
Absolute quantification for major bacteria genus on cecal samples by qPCR (copies/g sample). **(A)** Lactobacillus, **(B)** clostridium_sensus_stricto_1, and **(C)** Terrisporobacter. CON, control; CTC, chlortetracycline; XOS500, 500 mg/kg XOS; SEM, pooled standard error of the means. *P ≤ 0.05; **0.001 < *P* ≤ 0.01; ***P ≤ 0.001.

To determine absolute quantification of qPCR, nucleic acid standard was generated from genomic DNA of *Lactobacillus*, *Clostridium_sensu_stricto_1*, and *Terrisporobacter*. The C_*t*_-values were plotted as a function of the cell concentration and the plot showed the expected linear relationship between the copies per microliter (copies/μl) and C_*t*_-values (Supplementary Figure 4). Figure 6 showed that the absolute quantification of *Lactobacillus* in the XOS500 group was 7.23 × 10^9^ copies/g cecal sample and was approximately 2.6-fold higher than the CON group (approximately 2.01 × 10^9^ copies/g cecal sample). However, CTC supplementation remarkably reduced the absolute quantification of *Lactobacillus* (approximately 1.18 × 10^9^ copies/g cecal sample). In addition, the XOS500 group and CTC group markedly reduced the absolute quantification of *Clostridium_sensu_stricto_1* and *Terrisporobacter* compared with the CON group (*P* < 0.01).

### SCFA in the Cecum

**FIGURE 7:**
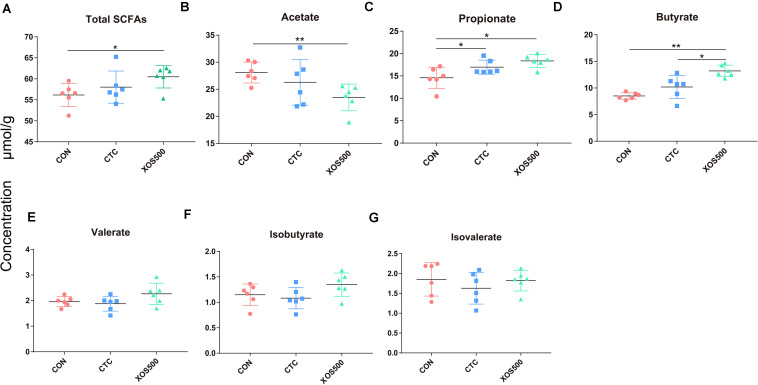
Short-chain fatty acids (SCFAs) concentration (μmol/g) in cecum of piglets during different dietary treatments. **(A)** Total SCFAs, **(B)** acetate, **(C)** propionate, **(D)** butyrate, **(E)** valerate, **(F)** isobutyrate, and **(G)** isovalerate. Total SCFAs are the sum of the following SCFAs: acetate, propionate, isobutyrate, butyrate, isovalerate, and valerate. Group differences were tested with a Duncan test. CON, control; CTC, chlortetracycline; XOS500, 500 mg/kg XOS. *P ≤ 0.05; **0.001 < *P* ≤ 0.01; ***P ≤ 0.001.

To further identify whether the observed microbial changes due to dietary treatment also affected the gut function, SCFA concentrations were measured. In the cecum, the most abundant SCFAs were acetate, propionate, and butyrate (Figure 7). Dietary supplementation with XOS500 significantly decreased the acetate concentrations and increased the total SCFAs, propionate and butyrate concentrations compared to the CON group in the cecal digesta (*P* < 0.05). In the CTC group, the concentration of propionate was remarkably higher than the CON group (*P* < 0.05). However, there is no significant difference in the SCFA concentrations between the XOS500 and CTC group.

## Discussion

In the present study, we evaluated the effect of the administration of CTC and XOS on the growth performance and intestinal health of weaned piglets. We used high-throughput sequencing of the V1-V3 region of the 16S rRNA gene to monitor the ileal and cecal microbiota of piglets fed either CTC or XOS. Furthermore, we detected the absolute quantification of the specific bacterial genera and measured SCFA concentration in the cecal sample. We showed that XOS consumption altered specific bacterial genera and fermentation metabolites. We hypothesize that the improvement in growth performance of piglets is related to the characteristics of the intestinal ecosystem. Previous studies have shown that XOS are good additives for improving animal growth performance. For example, Liu et al. (2018) reported that supplemented with XOS had a greater ADG and feed efficiency. Similarly, our results showed that dietary XOS500 supplementation had a positive effect on growth performance of weaned piglets compared with the CON group. Weaned piglets fed a diet supplemented with CTC exhibited greater performance than the CON group, but no differences were observed between the CTC and XOS500 group. Some broiler studies also showed that dietary XOS significantly increased the ADG and reduced FCR (De Maesschalck et al., 2015; Yuan et al., 2018). In addition, Pourabedin et al. (2015) found that feed conversion ratio (FCR) in broilers fed 2 g/kg XOS diets was significantly lower than those fed CTL or 1 g/kg XOS between days seven and 21. In contrast, Yin et al. (2019) reported that 0.01% XOS treatment failed to observe significant improvement in growth performance of the piglets. These reports indicate that different XOS doses may exert diverse effects on the growth performance of animals.

Intestinal morphology indices are often a useful criterion to estimate the nutrient digestion and absorption capacity of the intestine. Prebiotics were reported to improve growth *via* promoting nutrient absorption by improving intestinal structure (Pan et al., 2018). For example, Liu et al. (2018) demonstrated that XOS supplementation could improve the intestinal morphology and the apparent total tract digestibility of piglets. In this study, we found that villus height and villus height: crypt depth ratio of the ileum in the XOS500 treatment group was significantly increased compared to the CON group. Similarly, De Maesschalck et al. (2015) found that supplementation of 0.5% XOS to the broiler feed significantly improved the villus height of the ileum. Villi are important structures in the small intestine, mainly involved in nutrient absorption. Therefore, an increased villus height would increase the surface area for nutrient absorption (Choe et al., 2012). These results demonstrated that XOS may improve the gut absorptive function. The possible explanation for the improvement of intestinal morphology is that XOS500 supplementation stimulated the increase of *Lactobacillus*. Some studies found that *Lactobacillus* could improve the villus height and the villus height to crypt depth ratio of the ileum in the weaned piglets (Suo et al., 2012; Yi et al., 2018). Moreover, the numbers of goblet cells in the crypt of the cecum in the XOS500 treatment group was remarkably higher than the CON group. Goblet cells produce mucin glycoproteins, constituents of the mucus, which is in the first line of defense and protects the epithelium lining the intestinal mucosa from damage and invading pathogens (Strobel et al., 2015; Li et al., 2019). The increase of goblet cells numbers thus indicates that XOS500 supplementation contributes to an improvement of the chemical barrier (mucus layer) in the cecum. This effect on the large intestinal morphology may at least partly be due to the butyrate production improvement. In our study, the increasing of butyrate-producing genus *Blautia* and *Faecalibacterium* abundance in the hindgut may positively bring about butyrate production improvement. Therefore, XOS supplementation can be a promising approach for improving intestinal morphology and protecting the intestinal barrier function in pigs.

Great variations in α-diversity and β-diversity of the microbiota were found between the ileum and the cecum, similar to what has been previously observed in pigs (Zhang et al., 2018). Furthermore, the relative abundance of certain bacterial families or genera were differentially abundant in the ileum and cecum of the piglets. For example, the relative abundance of the family *Ruminococcaceae*, the genus *Blautia* and *Prevotella_9* was higher in the cecum compared with the ileum. The spatial changes in bacterial composition along the intestinal tract may result from the dramatic changes in the intestinal microenvironments. On the one hand, from the ileum to cecum, oxygen availability significantly decreases (Espey, 2013). On the other hand, pH gradient along the intestine is another important factor to influence the dynamic composition of the microbiota (Duncan et al., 2009). In addition, most dietary nutrients are fully digested at the end of the ileum under normal physiological conditions, and undigested material is then fermented by the microbiota in the large intestine.

High bacterial diversity is beneficial for the general health and productivity of animals (Lukić et al., 2019). We used the Chao1 index and Shannon index to compare the microbial diversity among different treatment groups. The CTC group both in the ileum and cecum showed a lower alpha diversity, which is in line with other studies using an antibiotic treatment (Knecht et al., 2014; Looft et al., 2014). Meanwhile, the CTC treatment significantly reduced the relative abundance of certain genera, mainly including *Lactobacillus* and *Clostridum_sensu_stricto_1* and *Escherichia-Shigella*. This observation is in accordance with the study of Zhang et al. (2016) who reported that CTC addition reduced the piglets *Escherichia-Shigella*, *Lactobacillus*, and *Streptococcus* abundance in the intestinal tract. In contrast, Kim et al. (2012) demonstrated that the relative abundance of the genus *Lactobacillus* increased due to the antimicrobial growth promoter tylosin exposure. In addition, our study showed that the CTC treatment markedly increased the propionate concentration compared with the CON group in the cecum. Previous research has also found that the concentration of propionate significantly increased as a result of the addition of antibiotic in broilers and was positively correlated with the change in the relative abundance of *Propionibacterium* (Pourabedin et al., 2015). As propionate is a well-known precursor of hepatic gluconeogenesis and is regarded as an inhibitor for lipogenesis, it thus seems that the improved animal performance of the CTC treatment is in part the result of the modulation of the microbiota.

The ileal and cecal microbiota was mainly composed of *Firmicutes*. Within this phylum, the majority belonged to the *Lactobacillus* genus which is consistent with previous 16S rRNA gene-based studies (Zhang et al., 2018). Our study found that the relative abundance of certain bacterial genera was altered with XOS500 supplementation in the ileum and cecum of the piglets. For instance, the relative abundances of *Lactobacillus* increased whereas *Clostridium_sensu_stricto_1* and *Escherichia_Shigella* decreased in the ileum. It was also noted that XOS500 supplementation significantly increased *Lactobacillus* level and reduced *Clostridium_sensu_stricto_1* and *Terrisporobacter* level by high-throughput sequencing of 16S rRNA gene amplicons in the cecum. Absolute quantification for these specific bacteria genera on cecal samples by qPCR further confirm the above results. Furthermore, this is in accordance with a recent study showing that XOS supplementation improved the *lactobacilli* abundance and reduced *Escherichia coli* abundance on d14 of weanling pigs (Liu et al., 2018). Similarly, a previous study reported that the cecal microbiota of HXOS-fed chickens contained significantly higher proportions of the *Lactobacillus* genus than the other dietary treatments (Pourabedin et al., 2015). Additionally, Christensen et al. (2014) also confirmed that XOS groups had higher relative abundance of *Lactobacillus* spp. than the CON group in rat cecum content. In contrast, Yin et al. (2019) found that the administration of XOS to piglets markedly reduced the relative abundance of the *Lactobacillus* genus. *Lactobacillus* is a dominant genus within the *Firmicutes* phylum having beneficial effects for health including the exclusion of pathogens, immunomodulation and the production of beneficial molecules (Kravtsov et al., 2008). The high abundance of *Lactobacillus* in the XOS500 group suggested that XOS has a real potential of promoting the proliferation of beneficial bacteria in the ileum and cecum. We hypothesize that the increase in *Lactobacillus* abundance may contribute to the improvement in the intestinal morphology and promote piglet growth. This observation is in accordance with the study of Yi et al. (2018) who reported that *Lactobacillus reuteri* LR1 improved the villus height to crypt depth ratio of the ileum in the weaned pigs. This positive relation between the increase of *Lactobacillus* population and improvement in weight gain has also been confirmed in other studies (Dumonceaux et al., 2006; Lin, 2011). The *Clostridium_sensu_stricto_1* genus has been shown to be correlated with epithelial inflammation in the intestinal mucosa (Wang et al., 2017). In addition, *Terrisporobacter* is a kind of emerging anaerobic pathogen and acetogenic bacterium, which can degrade various carbon sources, like xylose and cellobiose (Deng et al., 2015; Cheng et al., 2016; Groher and Weuster-Botz, 2016). Interestingly, our study showed that XOS500 supplementation only decreased the opportunistic pathogenic strains *Clostridium_sensus_stricto_1, Escherichia_Shigella* and *Terrisporobacter* and increased the beneficial bacteria genus *Lactobacillus*. However, CTC treatment not only reduced the abundance of pathogenic strains but also decreased the abundance of the beneficial bacteria. Therefore, the results further support that XOS500 supplementation may contribute to the resistance of piglets to disease and exert a protective effect on intestine as an alternative to in-feed antibiotics of weaned piglets for maintaining favorable gut microflora composition.

Some studies have shown that SCFAs, mainly acetate, propionate and butyrate produced by gut microorganisms have health-promoting effects. Acetate can be oxidized in the tricarboxylic acid (TCA) cycle or is involved in *de novo* lipogenesis by conversion into to acetyl-CoA, while propionate is a well-known precursor for gluconeogenesis in the liver and is regarded as an inhibitor for lipogenesis (Den Besten et al., 2013a). Butyrate is the favored energy source for large intestinal cells and the majority of this SCFA is absorbed and utilized within the large intestine (Wang R. X. et al., 2020), while acetate and propionate enter hepatic circulation in significant quantities (Den Besten et al., 2013b). Except energy provision, butyrate probably plays beneficial effects on gut morphology, growth performance and anti-inflammatory under normal physiological conditions (Carney, 2015; Huang et al., 2017) through regulation of gene expression like proinflammatory cytokines nuclear factor kappa B (NF-κB) and interferon gamma (IFN-γ) inhibition and activation of SCFA-specific G protein-coupled receptors (Klampfer et al., 2003; Layden et al., 2013; Silva et al., 2018). Our results showed that XOS500 supplementation greatly decreased the concentration of acetate. However, the concentrations of propionate and butyrate in the XOS500 group clearly increased. Consistent with these findings, previous studies also showed that dietary XOS significantly increased the butyric acid content and decreased the concentrations of acetate (Lecerf et al., 2012; Ding et al., 2017). A remarkable increase in cecal butyrate concentration was observed as a result of XOS500 treatment and was positively correlated with the change in the relative abundance of *Blautia* and *Faecalibacterium* in the cecum. Thus, the altered SCFA concentrations were closely associated with the changes in the intestinal microbiota. Taken the results of the intestinal morphology, microbiota and the SCFA together, it is suggested that XOS is an interesting alternative to antibiotics to promote growth performance and modulate gut health in weaning piglets. However, further research is needed on the detailed mechanism of XOS on the host gut microbiota.

## Conclusion

In conclusion, this study indicates that dietary XOS or CTC supplementation enhanced the growth performance, improved intestinal morphology and modulated the relative abundance of specific bacteria by changing the overall microbial structure and metabolites. The increased population of *Lactobacillus* and decreased abundance of *Clostridium_sensu_stricto_1*, *Escherichia_Shigella*, and *Terrisporobacte*r piglets fed XOS500 might be a growth-promoting attribute. Thus, XOS may potentially function as an alternative to in-feed antibiotics in weaned piglets in modern husbandry.

## Data Availability Statement

The datasets presented in this study can be found in online repositories. The names of the repository/repositories and accession number(s) can be found below: NCBI BioProject, accession no. PRJNA683772.

## Ethics Statement

The animal study was reviewed and approved by Animal Care and Use Committee of the Chinese Academy of Agriculture Sciences. Written informed consent was obtained from the owners for the participation of their animals in this study.

## Author Contributions

HZ and LC designed the experiments. YC drafted the manuscript. YC and YX carried out most of the experiments and analyzed the data. CL provides some help for animal feeding. RZ revised the manuscript. LL provided a guarantee for the smooth running of the entire trial. LX provided us XOS. HZ, LC, and NE had primary responsibility for the final content. All authors contributed to the article and approved the submitted version.

## Conflict of Interest

LX was employed by the company Shandong Longlive Bio-Technology Co., Ltd. The remaining authors declare that the research was conducted in the absence of any commercial or financial relationships that could be construed as a potential conflict of interest.

## Footnotes

1 http://qiime.org/index.html

2 http://drive5.com/uparse/

3 http://rdp.cme.msu.edu/
