# Supplementary material for: Effects of Xylo-Oligosaccharides on Growth and Gut Microbiota as Potential Replacements for Antibiotic in Weaning Piglets
Source: Front Microbiol. 2021 Feb 25;12:641172. doi: 10.3389/fmicb.2021.641172 (PMC7947891; doi:10.3389/fmicb.2021.641172)
Supplement: Supplementary file 1 [file Data_Sheet_1.docx]

**Supplementary Table S1** Primers used for absolute quantification of microbial populations in cecal digesta of weaned piglets.

| Item | Primer (5′-3′) | Annealing temperature (℃) | Product length (bp) |
| --- | --- | --- | --- |
| Lactobacillus | Forward: GAGGCAGCAGTAGGGAATCTTC | 60 | 118 |
|  | Reverse: CAACAGTTACTCTGACACCCGTTCTTC |  |  |
| Clostridium_sensu_stricto_1 | Forward: ATGCAAGTCGAGCGAKG | 55 | 120 |
|  | Reverse: TATGCGGTATTAATCTYCCTTT |  |  |
| Terrisporobacter | Forward: CGCAACCCTTGCCTTTAGT | 57.5 | 220 |
|  | Reverse: CCCTCTGTACCACCCATTGT |  |  |


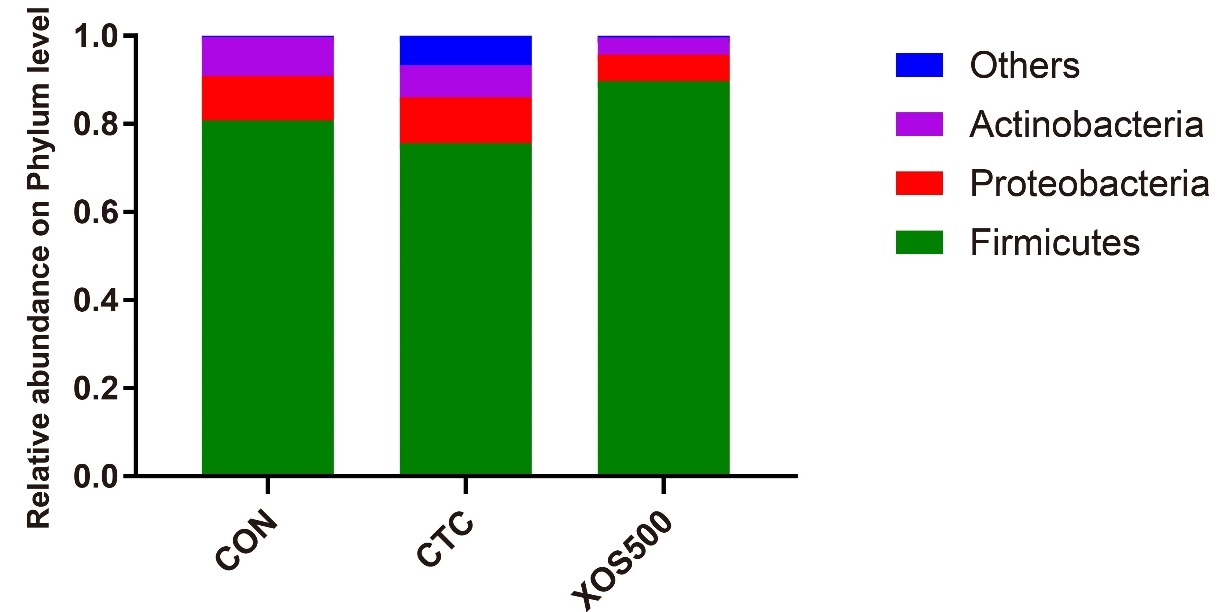


**Supplementary Figure S1** Characterization of the ileum microbiota at the phylum level. Kruskal-Wallis H test bar plot showed the major ileal bacterial phylum during the different treatment groups. CON: control; CTC: chlortetracycline; XOS500: 500mg/kg XOS.


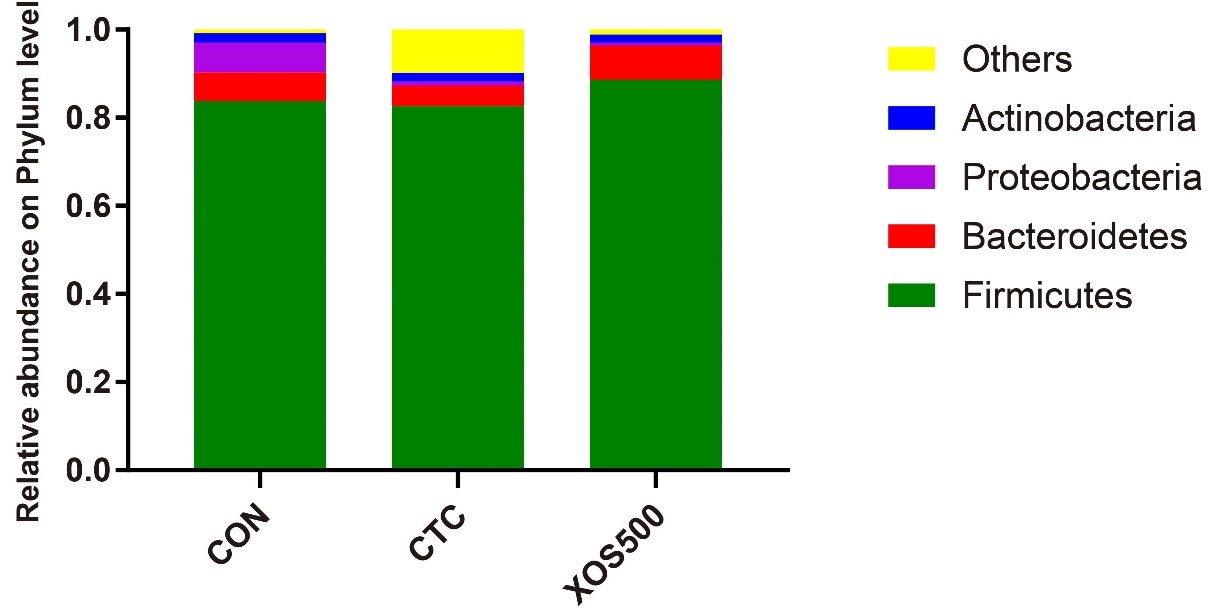


**Supplementary Figure S2** Characterization of the cecum microbiota at the phylum level. Kruskal-Wallis H test bar plot showed the major ileal bacterial phylum during the different treatment groups. CON: control; CTC: chlortetracycline; XOS500: 500mg/kg XOS.


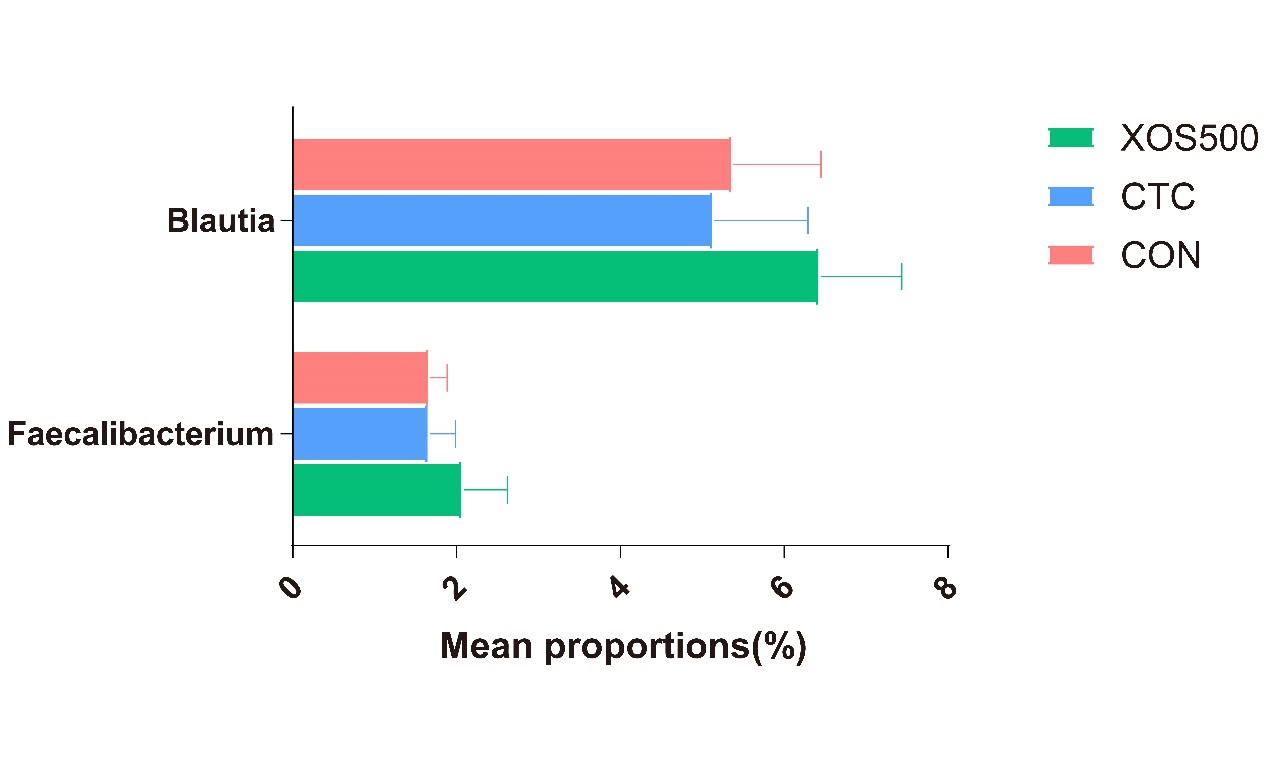


**Supplementary Figure S3** Kruskal-Wallis H test bar plot showed effect of dietary treatments on cecal Blautia and Faecalibacterium genus abundance of weaned piglets. CON: control; CTC: chlortetracycline; XOS500: 500mg/kg XOS.


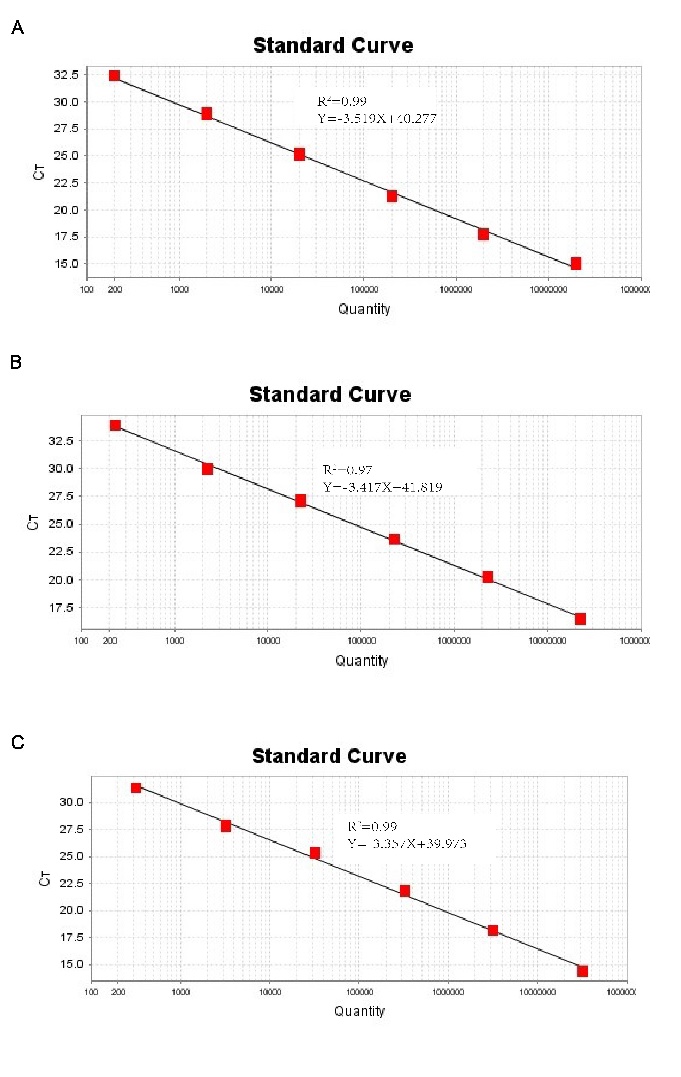


**Supplementary Figure S4** The standard curve for absolute quantification of specific bacteria in cecal sample. (**A**) Lactobacillus (**B**) Clostridum_sensus_stricto_1 (**C**) Terrisporobacter Standard curves produced from 10-fold serial dilutions ranging from 1×10^2^ to 1×10^8^ copies/ul cecal sample DNA, showing the relationship between C_t_-values and copies/ul for qPCR assays. C_t_, cycle threshold.
